# Hemoglobin and cytochrome *c*. reinterpreting the origins of oxygenation and oxidation in erythrocytes and in vivo cancer lung cells

**DOI:** 10.1038/s41598-023-41858-z

**Published:** 2023-09-07

**Authors:** Halina Abramczyk, Jakub Maciej Surmacki, Monika Kopeć, Karolina Jarczewska, Beata Romanowska-Pietrasiak

**Affiliations:** 1 Faculty of Chemistry, Institute of Applied Radiation Chemistry, Laboratory of Laser Molecular Spectroscopy, Lodz University of Technology, Wroblewskiego 15, 93-590 Lodz, Poland; 2 Oncological Surgery Department, Medical Genetics Department, Copernicus Provincial Multidisciplinary Centre of Oncology and Traumatology in Lodz, Pabianicka 62, 93-513 Lodz, Poland

**Keywords:** Cancer imaging, Cell biology, Imaging studies, Raman spectroscopy

## Abstract

Maintaining life (respiration), cell death (apoptosis), oxygen transport and immunity are main biological functions of heme containing proteins. These functions are controlled by the axial ligands and the redox status of the iron ion (oscillations between Fe^2+^ and Fe^3+^) in the heme group. This paper aims to evaluate the current state of knowledge on oxidation and oxygenation effects in heme proteins. We determined the redox status of the iron ion in whole blood (without and with anticoagulant), hemoglobin in erythrocytes, in isolated cytochrome *c* and cytochrome *c* in mitochondria of the human lung cancer cells using UV–VIS electronic absorption spectroscopy, Raman spectroscopy and Raman imaging. Here we discussed the mechanism responsible for the Q electronic absorption band spectral behavior, i.e., its splitting, and its change in extinction coefficient, as well as vibrational modifications upon oxygenation and oxidation. We compared the redox status of heme in hemoglobin of human erythrocytes and cytochrome *c* in mitochondria of human lung cancer cells. Presented results allow simultaneous identification of oxy- and deoxy-Hb, where 1547 and 1604 cm^−1^ vibrations correspond to deoxygenated hemoglobin, while 1585 and 1638 cm^−1^ correspond to oxyhemoglobin, respectively. Our results extend knowledge of oxidation and oxygenation effects in heme proteins. We demonstrated experimentally the mechanism of electronic-vibrational coupling for the Q band splitting. Presented results extend knowledge on oxidation and oxygenation effects in heme proteins and provide evidence that both processes are strongly coupled. We showed that retinoic acid affects the redox state of heme in cytochrome *c* in mitochondria. The change of the redox status of cytochrome *c* in mitochondria from the oxidized form to the reduced form has very serious consequences in dysfunction of mitochondria resulting in inhibition of respiration, apoptosis and cytokine induction.

## Introduction

Heme containing proteins are key proteins in maintaining life (respiration) and cell death (apoptosis), oxygen transport and immunity. Most of these functions are controlled by the axial ligands and the redox status of the iron ion in the heme group attached to the apoproteins. The oscillations between Fe^2+^ and Fe^3+^ can activate or inhibit these physiological functions. Hemoglobin is located in red blood cells (RBCs) (erythrocytes) consisting of four molecules: two alpha subunits and two beta subunits. Each subunit contains a heme group composed of a porphyrin ring with iron in the center. Each heme group binds one oxygen molecule, allowing each hemoglobin molecule to bind four oxygen molecules^1–3^.

The main function of hemoglobin (Hb) is to transport oxygen and remove carbon dioxide produced by the cells’ metabolism. Upon oxygen molecule binding as an axial ligand to the high-spin Fe (II) deoxyhemoglobin is converted to oxygenated hemoglobin. It is not clear if the iron Fe^2+^ is oxidized to Fe^3+^ by the bound oxygen and the reduction of O_2_ into O_2_‾. Some evidence indicates that oxyhemoglobin is a Fe^3+^ species, rather than Fe^2+^^4^.

The primary function of oxyhemoglobin (oxy-Hb) is to transport oxygen to cells and release molecular oxygen. As the oxygenated blood reaches cells and tissues, the oxygen is released from the hemoglobin of the blood and diffuses to the cells. Thus, the hemoglobin becomes deoxygenated (deoxy-Hb). Deoxygenated hemoglobin is in the Fe^2+^ ion ferrous reduced state and it changes the spin to low-spin *d*^6^ configuration.

Hemoglobin is partially susceptible to redox reactions where the functional Fe^2+^ ferrous heme is oxidized to Fe^3+^ ferric state and is known as methemoglobin. The oxidized hemoglobin loses its capacity to carry oxygen^5^. The methemoglobin concentration (metHb) in a healthy human subject does not normally exceed 1% of total circulating hemoglobin.

Cytochromes that are mainly located in the mitochondria transport electrons along the respiratory chain leading to the reduction of oxygen to water, and the storage of energy in the form of ATP. The transfer of electrons by cytochromes involves oxidation and reduction. During electron transfer, the iron atoms oscillate between the Fe^2+^ state and the Fe^3+^ state. Although the heme iron metal center changes oxidation state during the electron transport process, cytochrome *c* (Cyt *c*) always adopts an octahedral, low spin geometry regardless of the oxidation state on the iron^6^.

One cytochrome *c* molecule carries one electron, as the iron is reduced from ferric (Fe^3+^) to ferrous (Fe^2+^) form. The energy released in these reactions is captured as a proton gradient, which is then used to make ATP in a process called chemiosmosis. Together, the electron transport chain and chemiosmosis make up oxidative phosphorylation^7^.

We showed that cytochrome *c* plays a crucial role in the development and progression of cancer^8^.

Cytochromes *c* are ubiquitous heme proteins that are found in most living organisms and are essential for various energy production pathways as well as other cellular processes. Cytochrome *c* is a highly conserved ~ 12 kDa protein consisting of a single 104 amino acid peptide with four α-helices and a single heme group, which is covalently bound by two cysteine residues (Cys14 and Cys17)^9^.

In contrast to the heme of hemoglobin, cytochrome *c* does not attach oxygen as a ligand and as a result, the iron ion can be in both oxidation states, Fe^2+^ and Fe^3+^, and is in the low spin configuration. Cytochrome *c* binds to cardiolipin (CL) in the inner mitochondrial membrane, thus anchoring its presence and keeping it from releasing out of the mitochondria to initiate apoptosis in the cytoplasm^10^. Because of its ubiquitous nature and sequence homology, cytochrome *c* has been used as a model protein for testing molecular evolution^11^.

Cytochrome *c* plays a key role in the mitochondrial electron transport that shuttles electrons between respiratory complexes III and IV in mitochondria. Cytochrome *bc*_*1*_ from complex III donates one electron to the oxidized form of cytochrome *c* (Fe^3+^), reducing the iron of cytochrome *c* by one oxidation state to become Fe^2+^. The reduced cytochrome *c* may then be rapidly reoxidized by cytochrome *c* oxidase (complex IV) to regenerate oxidized cytochrome *c*. At normal physiological conditions, cytochrome *c* is mainly localized in the intermembrane space of the mitochondria. During pathological conditions, cytochrome *c* is released into the cytoplasm and triggers programmed cell death through apoptosis^12^. Cell death is an essential process for tissue development and renewal; however, excessive loss of cells can be a sign of disease.

We showed that cytochrome *c* can also be released into the extracellular space by damaged or dying cells and serve as an intercellular signaling molecule, possibly alerting the surrounding cells of tissue damage^13^. This extracellular function of cytochrome *c* in alerting surrounding cells and tissues of cellular damage and death makes cytochrome *c* similar to members of a diverse family of molecules termed damage-associate molecular patterns (DAMPs). As a result, cytochrome *c* is released into the bloodstream and is suggested to be a novel in vivo marker of mitochondrial injury^12^.

The cytochrome *c* favors its most common function as an electron shuttle between complexes III and IV of mitochondria, but the binding of cytochrome *c* to anionic phospholipids unfolds the protein and converts it from an electron shuttle into a potent peroxidase. In mitochondria, this peroxidase activity displays remarkable specificity towards cardiolipin, causing oxidation as well as hydrolysis^14^.

Cytochrome *c* in mitochondria changes its redox status between oxidized and reduced forms, but at the stationary Raman measurements at the normal physiological conditions both in tissue and in vitro cell cultures the oxidized form dominates^7^. This balance changes and is shifted towards reduced form in cancers. Cytochrome *c* operates at a low, basal level in normal cells, but it is strongly induced to very high levels in pathological cancer states^8^.

The redox status of central metal in heme proteins has created two long scientific debates: particularly concerned with the nature of the Fe-O_2_ bonding. The latter controversy has never disappeared dating back eight decades to the pioneering work of Pauling and is alive also today^15^.

From this point of view, therefore, it seems important to re-examine various aspects of the primary process in hemoprotein reactions with O_2_. This examination may provide us with further insights into general principles governing oxygen transport in hemoglobin and oxidative phosphorylation in cytochromes.

Advanced UV–VIS and Raman spectroscopy experiments may help in clarifying this issue as significant line shape differences between the protein-bound heme species reflect iron spin, redox state and oxygenation differences.

The aim of this paper is to answer the question of the mechanism(s) responsible for the Q electronic absorption band spectral behavior, i.e., its splitting, band broadening and its change in extinction coefficient, as well as vibrational modifications upon oxygenation and oxidation of the central iron ion of heme proteins in hemoglobin and cytochrome *c*. We studied hemoglobin inside human erythrocytes, cytochrome *c* in isolated form and in vitro human cells by using UV–VIS electronic absorption and Resonance Raman (RR) spectroscopy and Raman imaging. To study the processes of redox changes in cytochrome *c* in biological cells we recorded the Raman spectra of cells receiving redox stimuli by retinoic acid (RA) at in vitro cell cultures.

## Results and discussion

The mechanistic details of heme oxidation and oxygenation, which are of clinical, as well as of physical–chemical interest, have long been investigated by several authors, but a full understanding of these processes has not been reached so far^16–25^.

In this section, the results for whole human blood and isolated cytochrome *c* by using UV/VIS electronic absorption and for hemoglobin inside human erythrocytes and cytochrome *c* in human lung cancer cells by Resonance Raman spectroscopy and Raman imaging are presented. UV/VIS electronic absorption and RR spectroscopy and Raman imaging have long been applied to monitoring the properties of hemoglobin^16–21^ and cytochrome *c*^22–25^.

These techniques were used to study oxidation state, a spin of iron ion, oxygenation, quaternary structure (α_2_β_2_ tetramer), laser-induced oxidation, T and R states, air oxidation of whole blood, whole blood dried smears, erythrocytes, and packed erythrocytes^18,20,26–29^. The oxidation state, concentration and interactions with cardiolipin of cytochrome *c* in tissues, single cells, and isolated proteins have been studied^13,30–32^.

Although heme proteins including hemoglobin have been studied for many years, there are still many controversies regarding the effect of iron oxidation and oxygenation on the UV/VIS electronic spectra^15,32^.

**Figure 1:**
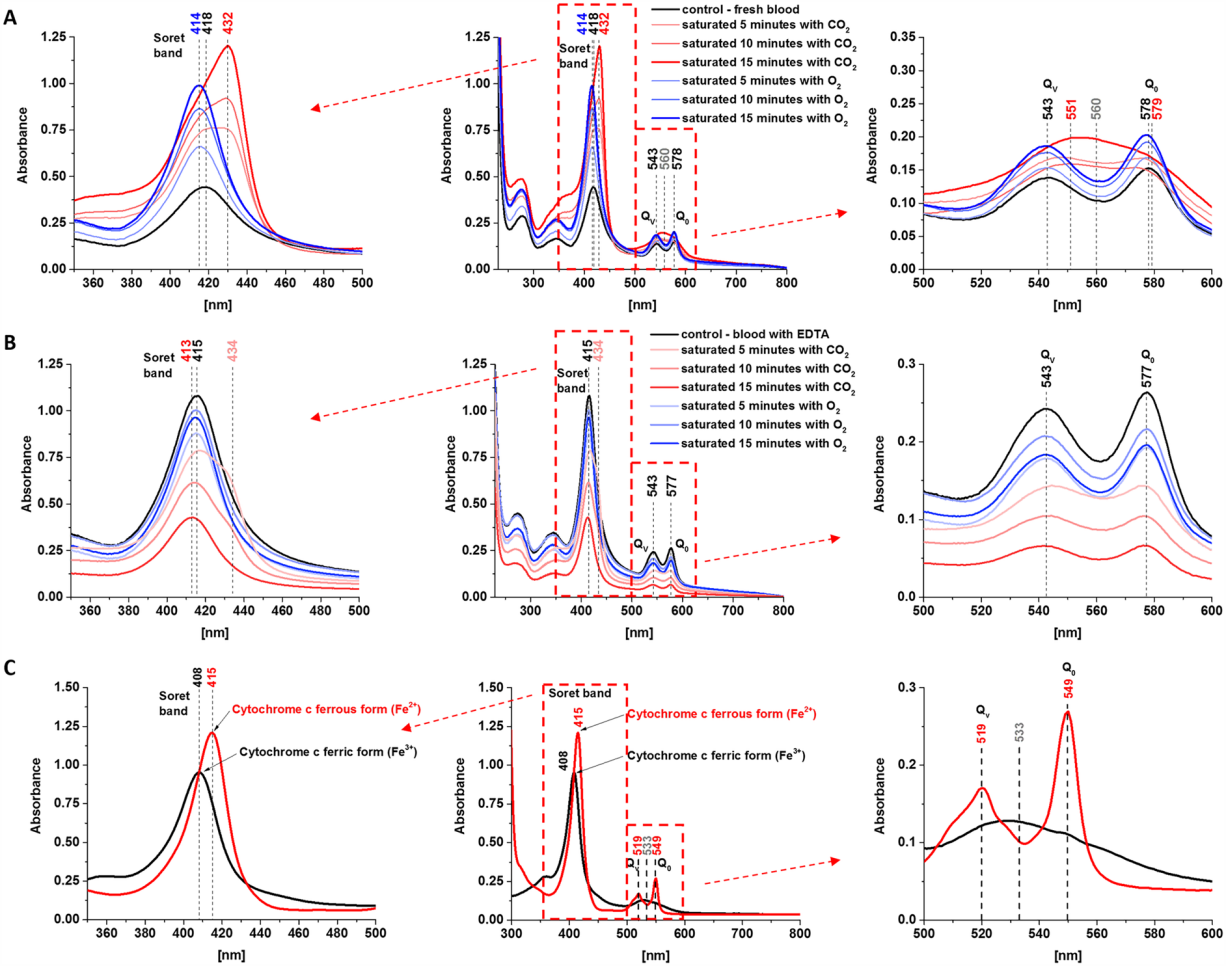
UV/VIS electronic absorption spectra of fresh whole human blood (**A**) and whole human blood with anticoagulant (ethylenediamine tetraacetic acid (EDTA)) (**B**), normal (control) and saturated with CO_2_ and O_2_, dissolved in potassium phosphate buffer, pH 7.4 and isolated cytochrome *c* (**C**) in ferric (Fe^3+^) and ferrous (Fe^2+^) forms. The cytochrome *c* sample (0.23 mM) was dissolved in potassium phosphate buffer, pH 7.4. The ferrous form of cytochrome *c* was prepared by adding tenfold excess of ascorbic acid as a reductant.

Figure 1 shows UV/VIS electronic absorption spectra of fresh whole human blood, whole human blood with anticoagulant (ethylenediamine tetraacetic acid, EDTA), hemoglobin inside the red blood cell (RBC) and cytochrome *c* in ferric Fe^3+^ (oxidized) and ferrous Fe^2+^ (reduced) states. Figure 1A shows UV/VIS electronic absorption spectra of fresh whole human blood (control) and blood saturated with CO_2_ and O_2_.

Figure 1A,B show the effect of axial ligation on the electronic absorption for oxygenated and deoxygenated hemoglobin at the same oxidation level Fe^2+^. The oxygenation and deoxygenation of hemoglobin were produced by saturation of the samples with O_2_ and CO_2_. One can see from Fig. 1A that for the fresh whole blood the intense Soret electronic transitions are centered: at 418 nm for the fresh whole blood (control), 414 nm for the whole blood saturated with O_2_ and 432 nm for the whole blood saturated with CO_2_. Saturation with O_2_ makes the blood more oxygenated, saturation with CO_2_ makes it less oxygenated. A detailed inspection of Fig. 1A shows that the band with a maximum at 432 nm consists of two overlapping bands, centered at 417 and 432 nm as obtained from the fitting of the band using the mixed function of Gaussian and Lorentzian (Table 1).

**Table 1:** A spectral band position indicated in UV–Vis spectra of fresh whole blood and with anticoagulant (normal and saturated with CO_2_ and O_2_) and isolated cytochrome *c* in ferric (Fe^3+^) and ferrous (Fe^2+^) forms.

	Soret band		Q band spectral splitting	
			Q_v_	Q_0_
Fresh whole blood				
Saturated with 15 min O_2_	414.43 ± 0.04	–	543.00 ± 0.30	578.00 ± 0.13
Saturated with 10 min O_2_	414.97 ± 0.04	–	543.00 ± 0.22	578.00 ± 0.10
Saturated with 5 min O_2_	415.61 ± 0.05	–	543.42 ± 0.11	577.49 ± 0.07
Normal (control)	417.68 ± 0.05	–	542.97 ± 0.13	578.18 ± 0.07
Saturated with 5 min CO_2_	417.05 ± 0.32	433.36 ± 0.13	546.53 ± 0.23	576.96 ± 0.18
Saturated with 10 min CO_2_	416.79 ± 0.42	432.48 ± 0.11	547.76 ± 0.14	577.53 ± 0.12
Saturated with 15 min CO_2_	416.51 ± 0.48	431.88 ± 0.09	551.32 ± 0.45	578.66 ± 0.39
Whole blood with EDTA				
Saturated with 15 min O_2_	414.36 ± 0.03	–	542.72 ± 0.08	577.04 ± 0.05
Saturated with 10 min O_2_	414.75 ± 0.03	–	542.99 ± 0.08	577.18 ± 0.05
Saturated with 5 min O_2_	414.78 ± 0.03	–	543.00 ± 0.12	577.50 ± 0.06
Normal (control)	415.20 ± 0.03	–	542.59 ± 0.11	577.23 ± 0.05
Saturated with 5 min CO_2_	416.54 ± 0.16	433.32 ± 0.09	543.00 ± 0.22	577.50 ± 0.16
Saturated with 10 min CO_2_	414.25 ± 0.06	433.71 ± 0.13	544.89 ± 0.11	576.66 ± 0.07
Saturated with 15 min CO_2_	412.74 ± 0.03	–	542.57 ± 0.09	576.51 ± 0.05
Cytochrome *c*				
Ferrous form (Fe^2+^, reduced)	–	415.29 ± 0.09	518.89 ± 0.21	549.35 ± 0.06
Ferric form (Fe^3+^, oxidized)	407.64 ± 0.09	–	529.40 ± 0.21	549.64 ± 0.25

For the whole blood with anticoagulant EDTA the Soret electronic transitions are centered at 415 nm for the control (without saturation) and for the sample saturated with O_2_. For the blood saturated with CO_2_, the Soret band at 434 nm is not as clear as for the fresh blood in Fig. 1A, but again it is evident from Fig. 1B that the Soret band consists of two overlapping bands centered at 415 and 434 nm as obtained from the fitting of the band (Table 1). The continuous shift of the maximum from 415 to 434 nm depends on the level of saturation with CO_2_ for 5, 10 min. However, for CO_2_ saturation for 15 min, the band at 434 nm disappears and only the band at 413 nm exists. Moreover, the signal intensity of the 413 nm band of the whole blood with anticoagulant EDTA decreases with the saturation level of CO_2_ in contrast to the whole fresh blood presented in Fig. 1A.

Figure 1C shows the effect of redox status on the electronic absorption of the ferric (Fe^3+^) and ferrous (Fe^2+^) forms of cytochrome. The effect of axial ligation is absent because cytochrome *c* which, in contrast to hemoglobin, is six-coordinated has two axial ligands: histidine and methionine Met80 that do not permit the iron center to interact with oxygen or CO_2_. Therefore, saturation with oxygen or CO_2_ does not produce oxygenation. Figure 1C shows the Soret band at 408 nm for the oxidized Fe^3+^ cytochrome *c*, and 415 nm for the reduced Fe^2+^ cytochrome *c* which is consistent with results published previously^18,21,32^.

In the view of results presented so far, we can summarize the electronic features of the Soret band. The bands at 418 nm and 415 nm represent reduced Fe^2+^ both for hemoglobin and cytochrome *c*, respectively. The slight blue shift from 415 to 408 nm in cytochrome *c* indicates the change of the redox status to the oxidized Fe^3+^ heme group in the protein. As one can see from Table 1 the slight blue shift from 418 to 414 nm is also observed for oxygenated hemoglobin saturated with O_2_, which may indicate that a small amount of blood exists in Fe^3+^ upon oxygenation and potential reduction of O_2_ into O_2_‾_._

In contrast to the slight shift upon oxidation of the central iron ion, the effect of the axial ligand (oxygen molecule) is significantly stronger. Figure 1A shows that the oxygenated hemoglobin in the fresh whole blood saturated with O_2_ shows a redshift from 414 to 432 nm when saturated with CO_2_. Saturation with O_2_ facilitates the binding of an oxygen molecule. The binding of a single oxygen molecule induces a conformational change that destabilizes the T (deoxygenated-tense) state and facilitates the transition of the other subunits to the high-affinity R (oxygenated-relaxed) state. Positive cooperativity in the binding of the first oxygen allows the second, third, and fourth oxygen molecules to subsequently bind with increasing ease^33^.

The decrease of the Soret band upon CO_2_ saturation observed in Fig. 1B may be related to the fact that oxygen binds axially to the iron atoms in the protein whereas carbon dioxide is bound to the protein chains of the structure. Thus, carbon dioxide doesn’t compete directly as a ligand with oxygen in this binding process in contrast to carbon monoxide. Despite the different ways of bonding to hemoglobin, saturation with CO_2_ results in deoxygenation. Saturation with CO_2_ produces carbaminohemoglobin, which in return stabilizes the T state, lowers affinity for oxygen, and induces oxygen unloading. There is a second channel that results also in deoxygenation. Upon entrance into red blood cells, carbon dioxide is quickly converted to carbonic acid by the enzyme carbonic anhydrase. Carbonic acid immediately dissociates into bicarbonate and hydrogen ions. As previously stated, an increase in hydrogen ions stabilize the hemoglobin in the T-state and induces oxygen unloading^34^.

The effect of oxidation and oxygenation is even better visible in the Q electronic transition. Our results in Fig. 1 show that the reduced Fe^2+^ cytochrome *c* and oxygenated reduced Fe^2+^ hemoglobin show evident spectral splitting in contrast to the oxidized cytochrome Fe^3+^ (Fig. 1C) and deoxygenated reduced Fe^2+^ hemoglobin in the whole blood (Fig. 1A) where only one broad band centered at 533 nm and 560 nm, respectively is observed. However, each of these bands at 533 nm and 560 nm consists in fact of two overlapping components as obtained from the fitting of the band (Table 1). The Q bands centered at 543 nm and 578 nm for the whole fresh blood and fresh blood saturated with O_2_, 543 nm and 577 nm for blood with EDTA, 519 nm and 549 nm for the reduced cytochrome *c*, 529 and 549 nm for the oxidized cytochrome *c,* 551 and 579 nm for deoxygenated reduced Fe^2+^ hemoglobin are observed (Table 1). UV–VIS spectra from Fig. 1 were fitted by using the mixed function of Gaussian and Lorentzian (defined method function: Gaussian-LorentzCross) in OriginPro software and spectral bands position are presented in Table 1.

Numerous studies have been performed to explore the influence of ligands and oxygenation, the oxidation and spin state of the central iron atom on the Q band spectral splitting^32,35,36^.

**Figure 2:**
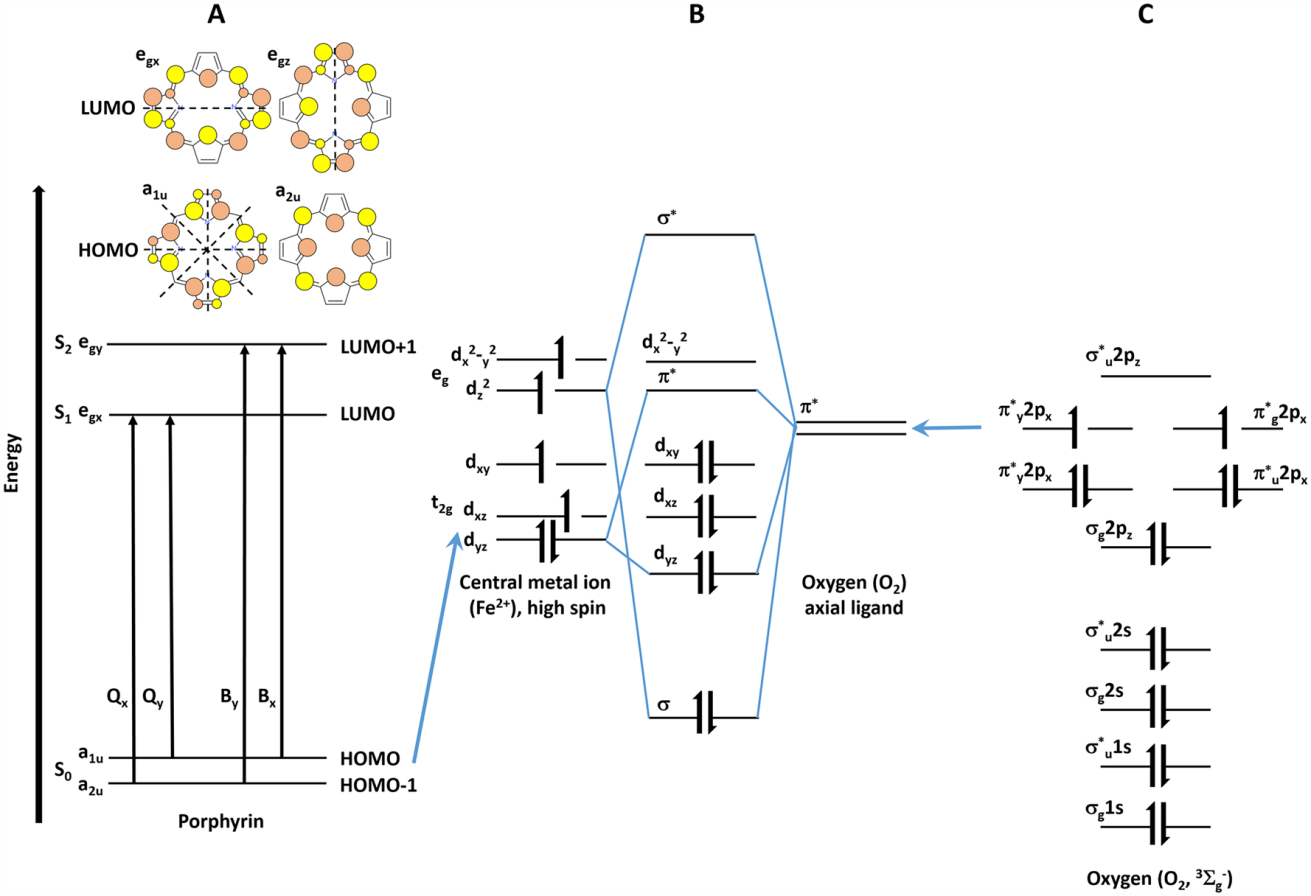
Schematic diagram of the energy levels of porphyrin, central metal atom and oxygen. (**A**) Gouteman’s four orbital model for a free-base state porphyrin, (**B**) molecular orbitals of high spin central metal ion (Fe^2+^) splitted by crystal field, (**C**) molecular orbitals of oxygen (O_2_).

The Soret and Q bands are rationalized by using Gouterman’s four-orbital model^37^ proposed to explain the absorption spectra of porphyrins allowed under D_4h_ symmetry, between the two highest occupied molecular orbitals (HOMO), a_1u_(π) and a_2u_(π), and the two degenerate lowest unoccupied orbitals (LUMO) (Fig. 2). Despite the symmetry-lowering factors caused by the insertion of the heme into a protein matrix, which effectively lowers this symmetry to C_4h_ or even C_2V_^38^, most theoretical calculations have been performed on square planar D_4h_ models^29^. The B and Q bands appear when the e_g_(π^*^) degeneracy is removed due to a geometrical distortion known as a Jahn–Teller effect^39^ that removes that degeneracy in the heme porphyrins. The Jahn–Teller effect is most often encountered in transition metal complexes described by ligands like oxygen, and crystal field theory (CFT).

The electronic transitions in metalloporphyrins including hemoglobin and cytochrome *c* are presented in Fig. 2. Soret band (B-band) in the UV/VIS spectral range arises from highly allowed (S_0_–S_2_) electronic transitions to the second excited state, and the weak Q-band in the visible spectral range arises from weakly allowed (S_0_–S_1_) electronic transition to the first excited state. Due to the mixing of states, the probability of transitions from S_0_ to S_2_ and from S_0_ to S_1_ is very different. The former is strongly allowed while the latter is only weakly allowed, resulting in dramatically different extinction coefficients for the two wavelengths corresponding to these two transitions.

Various interpretations have been proposed to account for lifting the e_g_(π^*^) degeneracy that results in the appearance of the B and Q bands. Generally, it has been suggested that heme-protein interactions and ligand-heme interactions lead to electronic perturbations^26,28,29,35,40^. The heme-protein interactions and ligand-heme interactions come from the metal (iron) center, the oxidation state of the heme iron, the substituents on the porphyrins ring and ligands (axial histidine and methionine in cytochrome *c*, axial histidine and O_2_ in oxygenated hemoglobin).

It is a common belief that interactions in metalloporphyrins including hemoglobin and cytochrome are dominated by electrons located in the conjugated system of carbon bonds in the porphyrin ring and by electrons of the transition metal ion located on the 3d orbitals. The overlap of the π molecular orbitals of the porphyrin ring with the dπ metal orbitals plays a significant role in determining the absorption spectrum of metalloporphyrins (Fig. 2). The electric field due to ligands removes the orbital degeneracy of the five 3d orbitals splitting them (typically) into lower-lying triplet (hybridized into t_2g_ orbitals) and higher energy doublet (hybridized into e_g_ orbitals) as described by ligands, and crystal field theory^39^. Depending on the energy difference between the triplet and the doublet levels, the ion can be either in high or low spin configuration. When the splitting between the triplet and doublet 3d orbitals is large (when oxygen is bound to the hemoglobin or when cytochrome *c* is oxidized), all six electrons of Fe^2+^ occupy the lower triplet, with the resulting spin equal to zero. When the splitting between the triplet and the doublet is small, the electrons occupy all five 3d orbitals, resulting in a high-spin configuration. The overlap between the π molecular orbitals of the porphyrin ring with the dπ metal orbitals is reflected in the slight shift of the Soret band from 415 nm for the ferrous Fe^2+^ to 408 nm ferric Fe^3+^ oxidation in cytochrome *c* (Fig. 1C). This type of interaction has also been used to explain the spectral behavior of the Q band^41^.

Another mechanism resulting from the heme-protein pocket for Q band splitting has been reported^32^. The Q band is thought to consist of two nearly degenerate electronic transitions in the excited state and the splitting is due to the asymmetry in the heme pocket of the protein created by the surrounding polypeptide chain. This asymmetry is thought to lead to the stabilization of one form of the excited state relative to the other, corresponding to a Jahn − Teller mechanism.

The interactions between the central ion and porphyrin ring, as well as the interactions between the heme protein pockets, are not sufficient to explain the effects of the ligands on Q band splitting, which are shown in Fig. 1A,B for fresh whole blood and whole blood with EDTA, respectively. The results presented here show a significant effect of the axial ligand (oxygen O_2_) on the UV/VIS absorption of the electronic transitions. Saturation with O_2_ and CO_2_ affects splitting of the Q transitions and the position of the doublet bands in the Q band. Figure 1A shows a change in the Q band from a single broad line (deoxyhemoglobin and oxidized cytochrome *c*) to a spaced doublet (oxyhemoglobin and reduced cytochrome *c*) (Fig. 1A,C). A detailed examination of Fig. 1A reveals the mechanism of this change. The absence of an axial O_2_ ligand in hemoglobin (high CO_2_ saturation 15 min) results in a broad band centered at 560 nm. Lower CO_2_ saturation (5 and 10 min) illustrates the mechanism of the Q band narrowing and splitting into two bands. When hemoglobin is more oxygenated by the axial ligand O_2_ (medium and low CO_2_ saturation, 10 min and 5 min) the splitting becomes visible at 543 nm and 578 nm. The splitting is clearly visible with oxygen-enriched hemoglobin (saturation for 5, 10 and 15 min with O_2_). This suggests that the mechanism of Q band splitting is due to decreasing band broadening, which could be partially explained by decreasing heterogeneity of heme proteins upon binding of the central iron ion with axial oxygen. However, a similar Q transition behavior is observed for cytochrome *c* upon a change in redox status (Fe^2+^/Fe^3+^), where oxygen is not bound to the central ion because the cytochrome is ligated by axial histidine and methionine.

**Figure 3:**
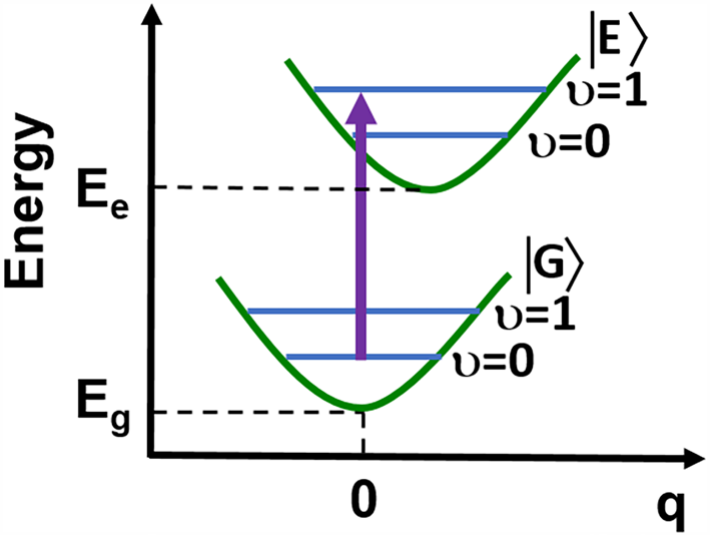
Vibrational coupling between the electronic and vibrational degrees of freedom in heme proteins.

Therefore, the measured Q-band splitting cannot be explained by electronic perturbations of the heme macrocycle alone, as they are unable to explain the ligand effect on Q-band spectral splitting. We suggest that the Q-band splitting (seen in Fig. 1 for oxygenated hemoglobin and reduced cytochrome *c*) is due to vibrational coupling between the electronic and vibrational degrees of freedom in heme proteins. We have shown that this mechanism dominates the spectral features in H-bond systems^42,43^, solvated electrons^44^ and bacteriorhodopsin^45,46^. In short, molecular excited states have geometries that differ from the ground state configuration, which is due to different electron configurations. The parametric dependence of the electronic energy on the nuclear configuration (q) leads to a variation in the electronic energy gap between states as one stretches the binding vibrations of the molecule. This effect is called vibrational coupling, which describes the coupling between electronic and nuclear degrees of freedom (Fig. 3).

The vibrational coupling has been reported to generate the vibronic bands Q_0_ and Q_v_ in heme proteins^29^. Based on the model of vibrational coupling, we assigned the Q_0_ transition to the lower energy Q transitions (578 nm for oxygenated fresh whole blood, 577 nm for oxygenated blood with EDTA, and 549 nm for reduced cytochrome *c*). The Q_v_ transition characterizes the higher energy transitions (543 nm for the oxygenated fresh whole blood, 543 nm for the blood with EDTA, and 519 nm for the reduced cytochrome *c*). In contrast, the single broad bands at 560 nm for the deoxygenated whole blood and at 533 nm for the oxidized cytochrome *c* in the mechanism of electron-vibrations coupling are due to the broadening of the Q_0_ and Q_v_ band because of vibrational dephasing caused by the heterogeneity of the electric field resulting from the interactions between the heme protein and ligand central ion and charged side chains in the protein environment. The assignments in Levantino et al.^29^ indicate that the Q_v_ band excited at 568, 531, 521 nm in resonance with Q transitions is mainly governed by the strong Herzberg-Teller coupling of A_2g_ vibrational modes. The spectra excited at 442 nm (near resonance with the Soret transition) show predominantly Raman bands of A_1g_ vibrational modes which gain intensity at this wavelength due to the Franck–Condon B state and Herzberg-Teller coupling.

To identify the corresponding heme vibrations coupled to the electronic degrees of freedom in the Q transition, we invoke the positions and intensities of the Raman peaks in the resonance Raman spectra recorded with 532 nm excitation.

Resonance Raman spectroscopy is a remarkably effective probe for the study of heme proteins in vitro cell cultures, tissues, and under in vivo conditions. Under nonresonant conditions cytochrome *c* (at a concentration of approximately 1 μM in the IMS (intramembrane space of mitochondria) or even less (nM))^47,48^ and hemoglobin in erythrocytes (at a concentration of approximately 5 mM)^49^ are obscured by stronger signals from other proteins, lipids and DNA. However, at the laser excitations corresponding to the electron resonances of the Soret or Q-band transitions, the Raman signal increases by several orders, and the Raman spectrum of cells and tissues is dominated by the vibrations of heme proteins.

**Figure 4:**
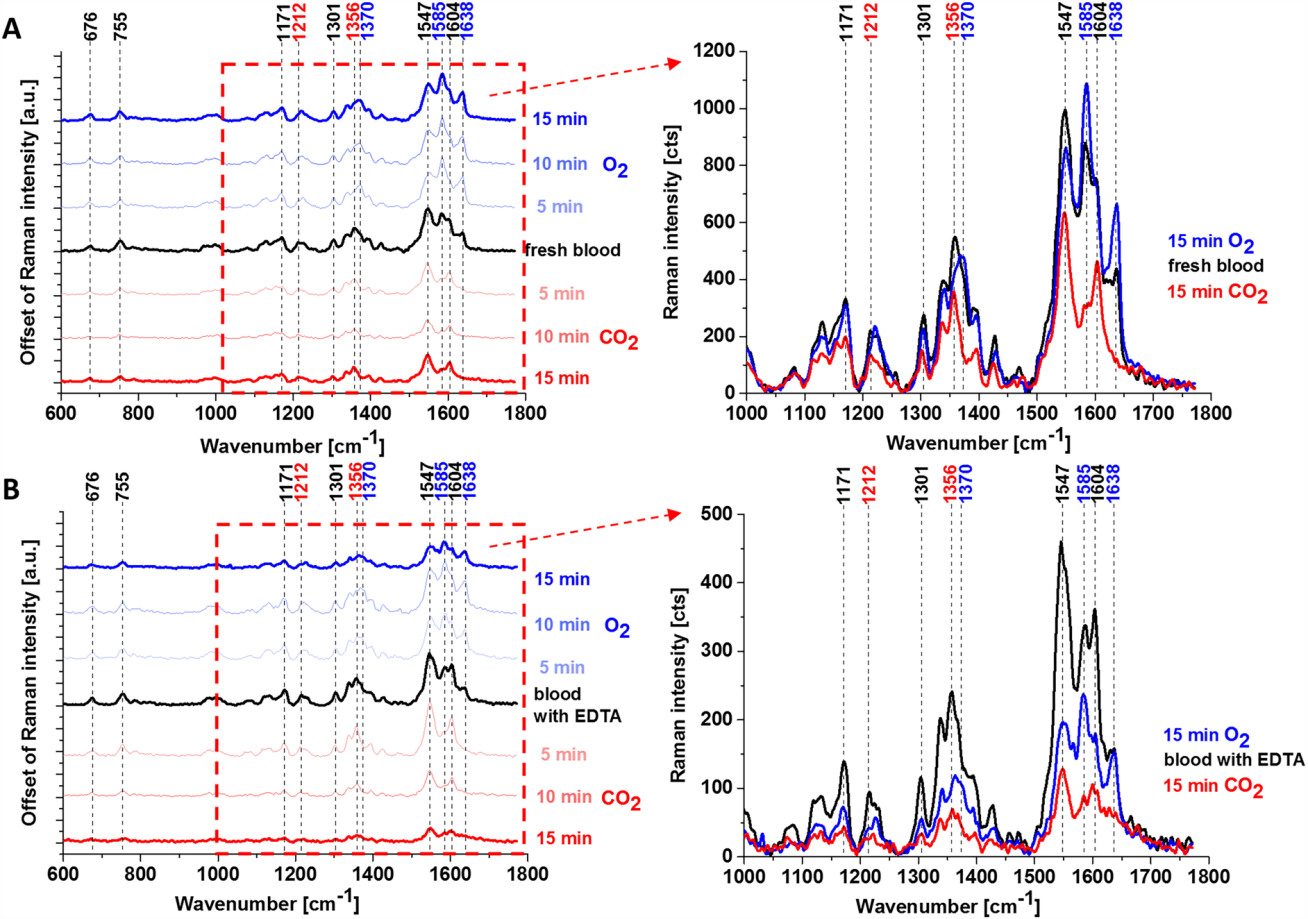
Raman spectra of fresh whole human blood (**A**) and whole human blood with anticoagulant (ethylenediamine tetraacetic acid (EDTA)) (**B**), normal and saturated with CO_2_ and O_2_.

Figure 4 shows Raman spectra of fresh human whole blood and hemoglobin inside the erythrocytes with EDTA saturated with CO_2_ and O_2_. The tentative assignments of Raman bands are presented in Table 2.

**Table 2:** A band position indicated in Raman spectra of fresh whole blood and with anticoagulant (normal and saturated with CO_2_ and O_2_).

Fresh whole blood (normal)	Fresh whole blood saturated with O_2_ (15 min)	Fresh whole blood saturated with CO_2_ (15 min)	Whole blood with EDTA	Whole blood with EDTA saturated with O_2_ (15 min)	Whole blood with EDTA saturated with CO_2_ (15 min)	Tentative assignments^18,21^
676	676	676	676	676	676	δ (pyr def)_sym_
755	755	755	755	755	755	ν(pyr breathing)
1171	1171	1171	1171	1171	1171	ν(pyr half-ring)_asym_
1212	–	1212	1212	–	1212	δ (C_*m*_H)
1301	1301	1301	1301	1301	1301	δ (C_*m*_H)
1356	–	1356	1356	–	1356	ν(pyr half-ring)_sym_
1370	1370	–	1370	1370	–	ν(pyr half-ring)_sym_
1547	1547	1547	1547	1547	1547	ν(C_ββ_)
1585	1585 ↑	1585 ↓	1585	1585 ↑	1585 ↓	ν(C_a_C_m_)_asym_
1604	1604 ↓	1604 ↑	1604	1604 ↓	1604 ↑	ν(C_a_C_m_)_asym_
1638	1638 ↑	–	1638	1638 ↑	–	ν(C_a_C_m_)_asym_

ν—stretching, δ—bending, def—deformation, sym—symmetric, asym—asymmetric, pyr—pyrrole, ↑—increase, ↓—decrease.

**Figure 5:**
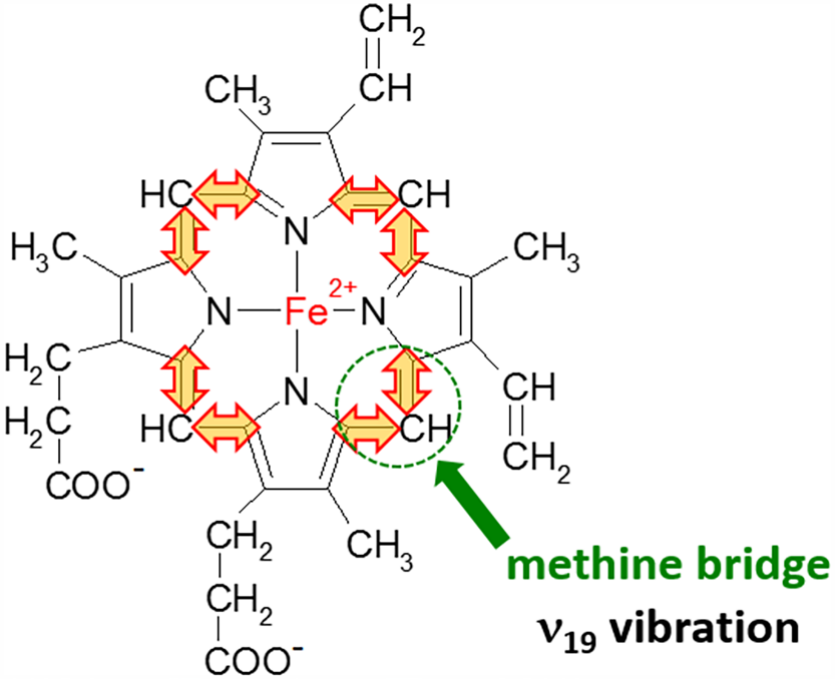
The vibration of methine bridge (band ν_19_ at 1585 cm^−1^).

Figure 4 shows that the Raman spectra taken with Q_0_-Q_v_-band excitation at 532 nm are dominated by bands from the asymmetric A_2g_ modes, i.e., 1585 cm^−1^ (ν_19_), 1604 cm^−1^ (ν_38_) and from the B_1g_ modes, i.e., 1638 cm^−1^ (ν_10_), 1547 cm^−1^ (ν_11_). The assignment of vibrations was taken from Spiro et al.^21^. We used the vibrational assignment reported by Hu et al.^50^ The other reports assigned the band at 1585 cm^−1^ to the ν_37_^20^. The band ν_19_ at 1585 cm^−1^ is primarily due to the methine bridge vibrations via C_α_–C_m_ stretching and C_m_–H bending modes (Fig. 5), respectively^21,51,52^. This vibration is formally equivalent to a rotation about the central z-axis, perpendicular to the heme plane.

A detailed inspection of Fig. 4 shows that the bands 1585, 1604, 1638, and 1547 cm^−1^ are very sensitive to oxygenation and can be regarded as Raman markers of oxygenation. The Raman intensity at 1585 and 1638 cm^−1^ increases with O_2_ saturation and decreases with CO_2_ saturation in contrast to 1547 and 1604 cm^−1^ vibrations.

This finding allows simultaneous identification of deoxy- and oxy-Hb, where 1547 and 1604 cm^−1^ vibrations correspond to deoxygenated hemoglobin, while 1585 and 1638 cm^−1^ correspond to oxyhemoglobin.

The symmetric modes A_1g_ at 1356 and 1370 cm^−1^ corresponding to ν_4_ vibration, which is strongly enhanced at Soret excitations, becomes much weaker at 532 nm excitation, but the effect of oxygenation is still clearly visible. Figure 4 shows that the band at 1370 cm^−1^ disappears at CO_2_ saturation and appears at O_2_ saturation. The 1356 cm^−1^ vibrations correspond to deoxygenated hemoglobin, while 1370 cm^−1^ corresponds to oxyhemoglobin. The ν_4_ band is due to an in‐phase breathing‐like mode of four pyrrole rings although being somewhat deformed by the large contribution of the C_α_–N symmetric stretching term^53^.

Our results from Fig. 4 support the earlier reports that band ν_4_ in hemoglobin is sensitive to the oxygenation level^49^.

**Figure 6:**
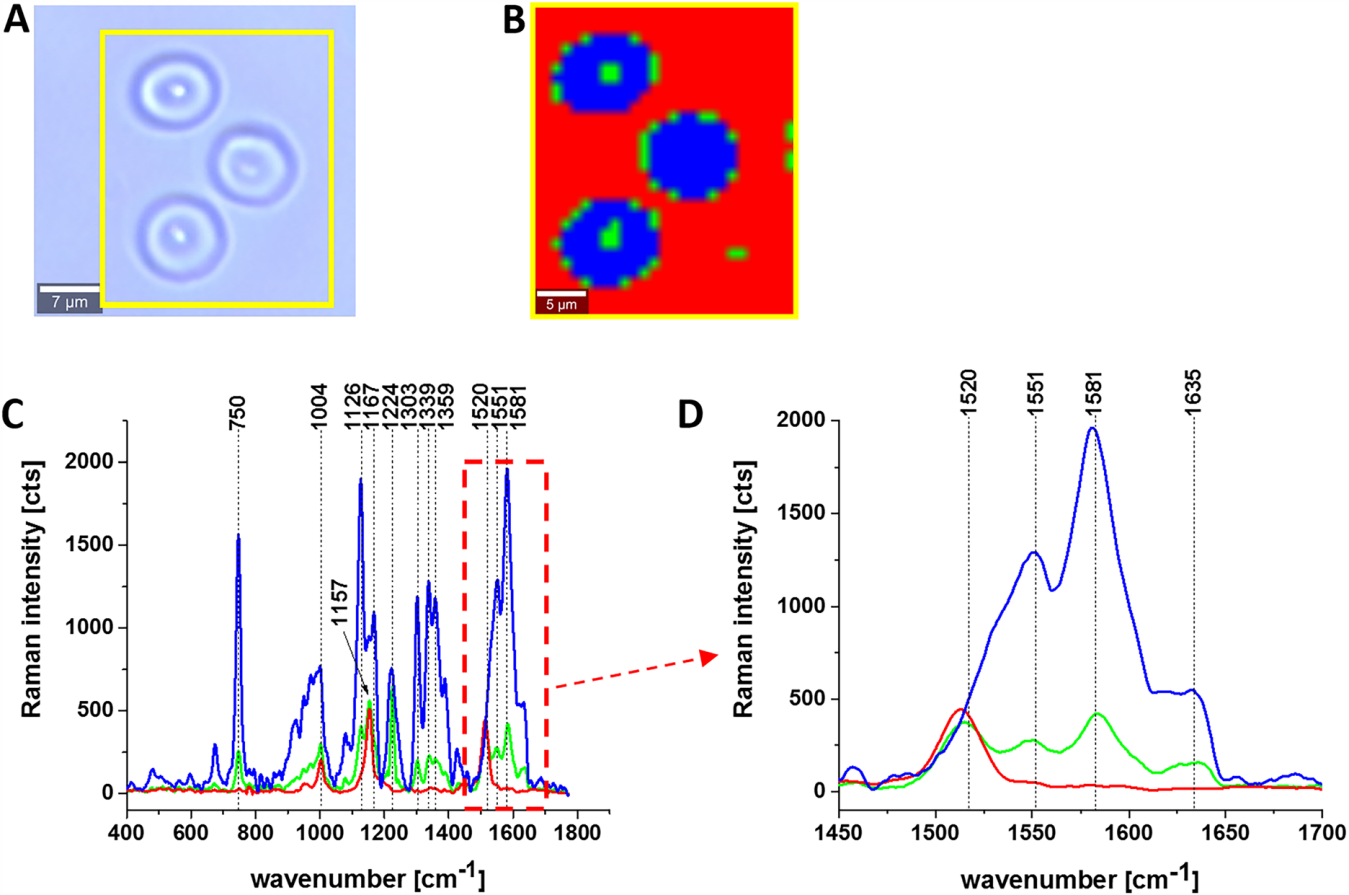
Raman imaging of hemoglobin in the red blood cells (RBC). (**A**) Microscope image, (**B**) Raman image and (**C**, **D**) Raman spectra of hemoglobin in RBC (dried smear). Resolution of image 1 μm, integration time 0.3 s, 3 mW at 532 nm.

Figure 6 shows the Raman images and spectra of erythrocytes (RBC) in the form of a dried smear.

The erythrocytes in dried smears presented in Fig. 6 come into long-term contact with air which may modify the properties of hemoglobin. However, comparing the results for the whole blood in Fig. 4 A with erythrocytes in the dried smears in Fig. 6 one can state that the results are similar and in both cases, the red blood cells are filled with a mixture of oxygenated Hb (1581 cm^−1^) and deoxygenated Hb (1551 cm^−1^). The red color in the Raman image and spectra at 1520 cm^−1^ and 1157 cm^−1^ correspond to carotenoids in plasma.

So far we studied the effect of the axial ligand from oxygen on the Raman spectra of hemoglobin. In this case, one can separate the effect of oxygenation from other interactions, particularly the redox status of the central metal ion, because both deoxygenated and oxygenated hemoglobin are in the same Fe^2+^ ion ferrous reduced state.

Now, we will concentrate on the influence of the redox status of the central iron ion on the Raman vibrations of heme proteins. We have chosen cytochrome *c* because it does not attach oxygen. Therefore, one can separate oxygenation from redox changes Fe^2+^/ Fe^3+^.

**Figure 7:**
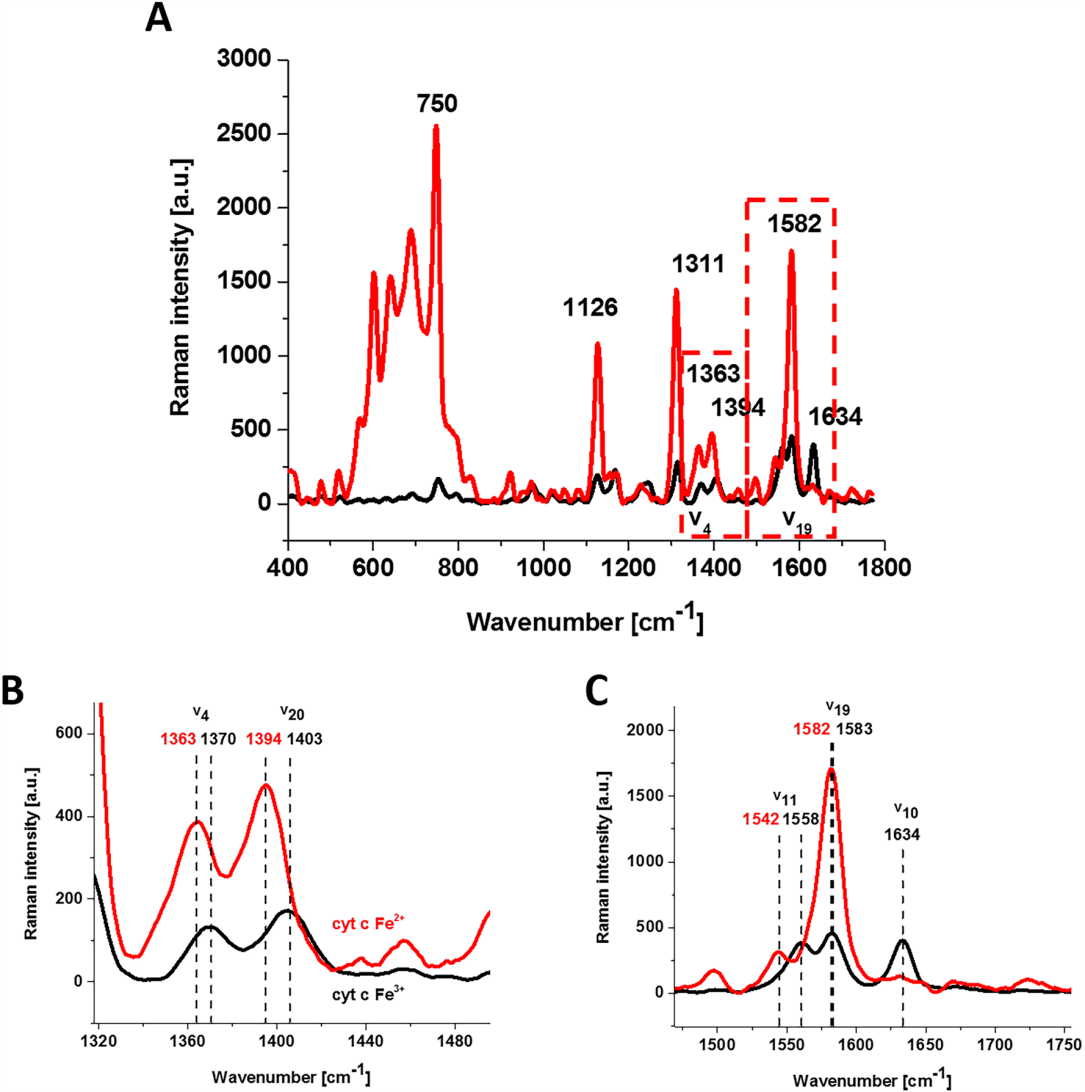
Raman spectra of isolated cytochrome *c* in solution (0.23 mM) dissolved in potassium phosphate buffer, pH 7.4, oxidized ferric Fe^3+^ (black line) and reduced ferrous Fe^2+^ (red line) cytochrome *c* (**A**) and an enlargement of the spectral region of 1318–1495 cm^−1^ (**B**) and 1470–1755 cm^−1^ (**C**). Ferrous cytochrome *c* was prepared by adding tenfold excess of reductor ascorbic acid.

Figure 7 shows Raman spectra of oxidized and reduced cytochrome *c*.

From Fig. 7A, it can be seen that the reduced cytochrome has a much higher intensity of Raman bands. The Raman band ν_19_ of cytochrome *c* corresponding to the methine bridge vibrations is only slightly sensitive to the oxidation state of iron the ion and appears at 1582 cm^−1^ for the reduced cytochrome *c* and at 1583 cm^−1^ for the oxidized one. In contrast, the Raman band ν_11_ of cytochrome *c* is sensitive to the oxidation state of the iron ion and appears at 1542 cm^−1^ for the reduced Fe^2+^ cytochrome and 1558 cm^−1^ for the oxidized Fe^3+^ cytochrome *c*.

The B_1g_ mode, i.e., 1634 cm^−1^ (ν_10_), which is relatively strong in comparison with the other vibrations of the oxidized cytochrome *c* disappears for the reduced cytochrome *c*.

From Fig. 7B, it is evident that the Raman band ν_4_ of cytochrome *c* is sensitive to the oxidation state of the iron ion, appearing at 1370 for the reduced Fe^2+^ cytochrome and at 1363 cm^−1^ for the oxidized Fe^3+^ cytochrome *c*. This trend is similar for the other heme proteins with the ν_4_ position either between 1356 and 1361 cm^−1^ for reduced (ferrous) heme proteins, and between 1370 and 1378 cm^−1^ for oxidized (ferric) proteins^19,54^. It is interesting to note that the shift from reduced to oxidized cytochrome (1363 cm^−1^ → 1370 cm^−1^) is very similar to that observed for the oxygenation effect in hemoglobin with the shift from deoxygenated to oxygenated hemoglobin (1356 cm^−1^ → 1370 cm^−1^). This could indicate that the oxygenation processes are coupled with the redox changes of the central iron ion. It is evident from Fig. 7B that the Raman band ν_20_ of cytochrome *c* is also sensitive to the oxidation state of the iron ion and appears at 1394 cm^−1^ for the reduced Fe^2+^ cytochrome and 1403 cm^−1^ for the oxidized Fe^3+^ cytochrome *c*.

So far, we have compared Raman spectra of hemoglobin in functional erythrocytes with the spectra of isolated cytochrome *c*. Now, we want to focus on the redox status of cytochrome *c* in the specific organelles within single cells in vitro. For this purpose, we incubated A549 with retinoic acid (RA) at concentrations of 1, 10 and 50 μM for 24 and 48 h of incubation.

**Figure 8:**
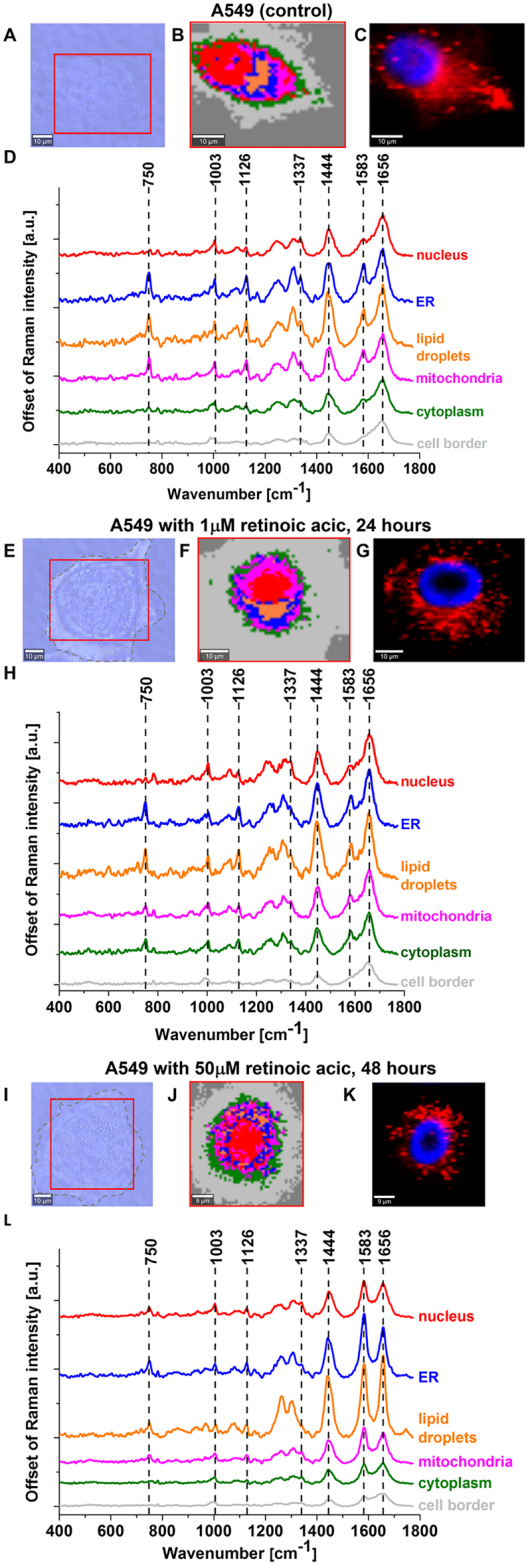
Raman imaging of lung cancer cells (A549) control and supplemented with 1 and 50 μM retinoic acid by 24 and 48 h. (**A**, **E**, **I**) Microscopy images, (**B**, **F**, **J**) Raman images with respective Raman spectra (**D**, **H**, **L**) of a nucleus (red), lipid droplets (orange), endoplasmic reticulum (blue), cytoplasm (green), mitochondria (magenta), cell border (light grey) and fluorescence images (**C**, **G**, **K**) (nucleus (blue, Hoechst 33,342), lipid-rich organelles (red, Oil Red O). Resolution of images 1 μm, integration time 0.3 s, 10 mW at 532 nm for Raman imaging.

Figure 8 shows the imaging analysis of a typical lung cancer cell (A549) control and supplemented with 1 and 50 μM retinoic acid for 24 and 48 h, respectively, for nucleus, lipid droplets, endoplasmic reticulum, cytoplasm, mitochondria and cell border by Raman spectroscopy.

**Figure 9:**
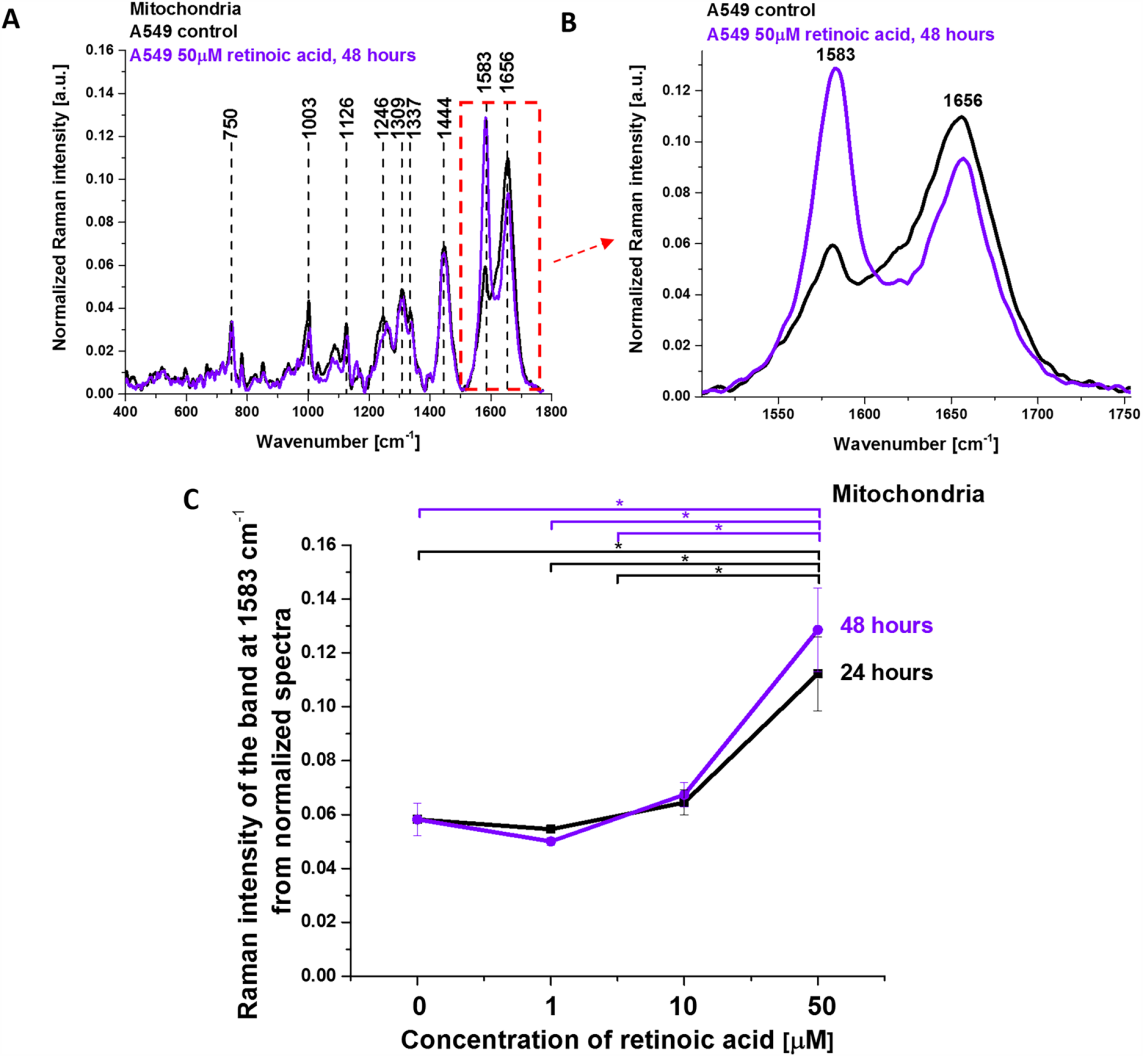
Average Raman spectrum of mitochondria in lung cancer cells (A549) incubated with retinoic acid (50 µM, incubated for 48 h). (**A**,**B**) Average Raman spectra (normalized by norm) were obtained from 3 cells. (**C**) Raman band ν_19_ intensity at 1583 cm^−1^ as a function of retinoic acid concentration (0–50 μM) in the mitochondria of a lung cancer cell. The statistically significant (*p* < 0.05) results have been marked with an asterisk.

Figure 9 shows the average Raman spectrum of mitochondria in human lung cancer cells (A549) incubated with 50 µM retinoic acid for 48 h and Raman band ν_19_ intensity at 1583 cm^−1^ as a function of retinoic acid concentration (from 0 to 50 µM, incubated for 24 and 48 h) in the mitochondria of a lung cancer cell. The band at 1583 cm^−1^ corresponds to the heme group of cytochrome *c*, the band at 1656 cm^−1^ represents the Amide I vibration of proteins in the mitochondria of the cell. For comparison, Fig. 9 shows also the control Raman spectrum without retinoic acid. One can see a dramatic increase in the Raman signal of the ν_19_ band at 1583 cm^−1^ upon incubation with retinoic acid. This enhancement illustrates the change of the redox status of the heme group from the oxidative iron ion Fe^3+^ to Fe^2+^ as we showed in Fig. 7A that the Raman bands of the reduced form of cytochrome *c* have much higher intensities than those of the oxidized form.

Figure 9C shows Raman band ν_19_ intensity at 1583 cm^−1^ as a function of retinoic acid concentration. One can see that at low concentrations of retinoic acid, up to 10 µM the intensity of the Raman signal in mitochondria does not change indicating that the cytochrome *c* is in the oxidized state with Fe^3+^ iron ion of the heme group. For higher concentrations of retinoic acid Raman signal increases spectacularly, which illustrates a change in the redox status of cytochrome *c* from the oxidized Fe^3+^ to the reduced Fe^2+^ state.

Our results show that retinoic acid affects the redox state of heme in cytochrome *c* in mitochondria. The change of the redox status in mitochondria from the oxidized form to the reduced form as a result cytochrome *c* cannot activate the apoptosome and initiate apoptosis and cytokine induction. In other words, it cannot play the role of a universal DAMP molecule able of alarming the immune system of danger in any type of cell or tissue by aborting the activation of the conventional mitochondrial signaling pathways^55^.

## Conclusions

This work has helped to resolve the longstanding controversy concerning the iron-oxygen interaction and metal spin state in heme proteins. UV–VIS spectroscopy combined with Raman spectroscopy and imaging provides an excellent tool to extend our knowledge of oxidation and oxygenation effects in heme proteins. We studied the effect of the redox status of the central iron ion and axial ligand (oxygen) in heme proteins inside erythrocytes of human blood and human lung cancer cells. We determined the effect of axial oxygen and the redox status of hemoglobin in erythrocytes, in isolated cytochrome *c* and cytochrome *c* in mitochondria of the lung cancer cells. We showed that the Q band splitting mechanism is governed by the vibrational coupling between electronic and vibrational degrees of freedom. Both the redox status of the central iron (Fe^2+^ or Fe^3+^), as well as the axial ligand (oxygen), are responsible for vibrational band broadening (vibrational dephasing), which affects the Q band splitting due to vibrational coupling. The vibrational coupling in hemoglobin and cytochrome *c* is dominated by the asymmetric A_2g_ mode corresponding to methine bridge vibrations (1585 cm^−1^ (ν_19_)). Our results allow simultaneous identification of oxy- and deoxy-Hb, where 1547 cm^−1^ and 1604 cm^−1^ vibrations correspond to deoxygenated hemoglobin, while 1585 and 1638 cm^−1^ correspond to oxyhemoglobin. The methine bridge vibrations at 1585 cm^−1^ are less sensitive to the charge of the central iron ion (Fe^2+^ or Fe^3+^). The Raman band ν_19_ of cytochrome *c* is only slightly sensitive to the oxidation state of iron ion and appears at 1582 cm^−1^ for the reduced Fe^2+^ cytochrome and 1583 cm^−1^ for the oxidized Fe^3+^ cytochrome *c*. The pyrrole ring vibrations of the macrocycle are more sensitive both to the effect of the redox state of iron ion and the axial ligand. The band at 1370 cm^−1^ appears at O_2_ saturation and disappears at CO_2_ saturation. The 1356 cm^−1^ vibrations correspond to deoxygenated hemoglobin, while 1370 cm^−1^ corresponds to oxyhemoglobin. The Raman band ν_4_ of cytochrome *c* is also sensitive to the oxidation state of iron ion and appears at 1370 for the reduced Fe^2+^ cytochrome *c* and 1363 cm^−1^ for the oxidized Fe^3+^ cytochrome *c*.

We showed that retinoic acid affects the redox state of heme in cytochrome *c* in mitochondria. The change of the redox status of cytochrome *c* in mitochondria from the oxidized form to the reduced form has very serious consequences in the dysfunction of mitochondria resulting in inhibition of respiration, apoptosis and cytokine induction. Therefore, cytochrome *c* cannot play the role of a universal DAMP molecule able of alarming the immune system of danger in any type of cell or tissue by aborting the activation of the conventional mitochondrial signaling pathways.

## Materials and methods

### Ethics statement

Blood samples were obtained from the Voivodeship Multi-Specialist Center for Oncology and Traumatology in Lodz. The spectroscopic analysis did not affect the scope of the course and type of hospital treatment undertaken. Written informed consent was obtained from all patients, or if subjects are under 18, from a parent and/or legal guardian. The Bioethical Committee at the Medical University of Lodz, Poland (RNN/17/20/KE) approved the measurement protocols. All the experiments were carried out in accordance with Good Clinical Practice and with the ethical principles of the Declaration of Helsinki.

### Reference chemicals

Cytochrome *c* (no. C2506) and retinoic acid (no. R2625) were purchased from Merck Life Science.

### RBCs sample

For all experiments, we used human fresh blood obtained from 24 healthy donors. Two types of blood samples were analyzed: whole blood and blood with ethylenediamine tetraacetic acid (EDTA anticoagulant). Samples with EDTA were subsequently centrifuged at 3500 rpm for 5 min at 18 °C to separate specific cell types into three layers: the top layer of plasma, the middle white layer composed of white blood cells and platelets, and the bottom red layer composed of red blood cells.

### UV–Vis spectrophotometry (UV–Vis)

Absorption spectra were recorded on a Perkin Elmer Lambda 750 spectrometer in the range of 230–800 nm using a quartz cuvette of a 1 mm path length. By UV–Vis spectrophotometry, we analyzed diluted RBC (50 μl RBC in 450 μl phosphate-buffered saline (PBS, no. 10010023, Gibco)). UV–VIS spectra were fitted by using the mixed function of Gaussian and Lorentzian (defined method function: Gaussian-LorentzCross) in OriginPro software.

### Raman spectroscopy and imaging

Raman measurements of the functional RBCs fractions obtained from the fresh blood were conducted on a WITec confocal Alpha 300RSA Raman microscope with the use of 532 nm excitation wavelength coupled to the microscope via an optical fiber (50 μm diameter). The 40 × objective (NIKON CFI Plan Fluor C ELWD (Extra-Long Working Distance) 40 × : N.A. 0.60, W.D. 3.6–2.8 mm; DIC-M, C.C.0–2) was used. A standard alignment procedure (single-point calibration) was performed before the collection of Raman spectra with the use of Raman scattering vibration produced by a silicon plate (520.7 cm^−1^).

For Raman imaging, a 10 μL drop of the red blood cells was placed on clean CaF_2_ windows (Crystran) and measured as a dried smear. For Raman spectra, we analyzed diluted RBC (50 μl RBC in 450 μl phosphate-buffered saline (PBS, no. 10010023, Gibco)) in a 1 mm quartz cuvette. The spectra were measured with a 532 nm excitation wavelength laser with the power of 3 mW for RBC smear (Raman imaging) or 10 mW in solutions in the focus spot. No damage to the sample was identified. Raman spectra were collected from at least ten randomly chosen spots (line scan mode: integration time 0.5 s and 10 accumulations). Raman data were analyzed using WITec (WITec Project Plus 4) and OriginPro 2018 programs. Preprocessing of Raman spectra included Cosmic Ray Removal (CRR; filter size: 4; dynamic factor: 4) and Background Subtraction (BG; polynomial order: 3). Raman imaging data were analyzed by the Cluster Analysis method described in our previous papers^13,30^.

### Statistical analysis

The statistical significance of the mitochondrial Raman band at 1584 cm^−1^ was analyzed using an ANOVA test (Origin Pro, OriginLab Corporation, Northampton, USA); p < 0.05 was considered statistically significant.

### Cell culture and preparation for Raman spectroscopy

An A549 human lung carcinoma cell line (no. CCL-185, ATCC) was used. A549 cells were grown in F-12K Medium (no. 30-2004, ATCC) with 10% fetal bovine serum (FBS no. 30-2020, ATCC) and maintained at 37 °C in a humidified atmosphere containing 5% CO_2._ Cells were seeded on a CaF_2_ window (Crystran Ltd., Poole, UK; CaF_2_ Raman grade optically polished window 25 mm diameter × 1 mm thick, no.CAFP25-1R, Poole, UK) in a 35 mm Petri dish at a density of 5 × 10^4^ cells per Petri dish the day before the examination. Before Raman examination, cells were supplemented with retinoic acid (1, 10, and 50 μM) for 24 and 48 h, then fixed with 4% formalin solution (neutrally buffered) and kept in PBS (no. 10010023, Gibco) during the experiment. For each culture condition, at least 3 cells were imaged with a minimum number of Raman spectra of 1600 for a single cell.

### Cell culture staining for fluorescence imaging

Fluorescence staining was performed after Raman imaging by using Hoechst 33,342 (25 μL at 1 μg/mL per mL of PBS; images collection: excitation: 355 nm, integration time: 0.01 s, resolution: 1 μM) and Oil Red O (10 μL of 0.5 mM Oil Red dissolved in 60% isopropanol/dH_2_O per each mL of PBS, excitation: 532 nm, integration time: 0.01 s, resolution: 1 μM) dyes by incubation for 15 min. The cells were imaged for fluorescence using a WITec Alpha 300RSA microscope.

## Data Availability

The datasets used and analyzed during the current study are available from the corresponding author on reasonable request.
